# Rescue of an aggressive female sexual courtship in mice by CRISPR/Cas9 secondary mutation in vivo

**DOI:** 10.1186/s13104-018-3307-8

**Published:** 2018-03-27

**Authors:** Jozsef Zakany, Denis Duboule

**Affiliations:** 1 Department of Genetics and Evolution, University of Geneva, Geneva, Switzerland; 2 School of Life Sciences, Ecole Polytechnique Fédérale, Lausanne, Switzerland

**Keywords:** Social behavior, Hippocampus, *HOXD10*, Genome editing, CRISPR/Cas9

## Abstract

**Objective:**

We had previously reported a mouse line carrying the *Atypical female courtship* (*HoxD*^*Afc*^) allele, where an ectopic accumulation of *Hoxd10* transcripts was observed in a sparse population of cells in the adult isocortex, as a result of a partial deletion of the *HoxD* gene cluster. Female mice carrying this allele displayed an exacerbated paracopulatory behavior, culminating in a severe mutilation of the studs’ external genitals. To unequivocally demonstrate that this intriguing phenotype was indeed caused by an illegitimate function of the HOXD10 protein, we use CRISPR/Cas9 technology to induce a microdeletion into the homeobox of the *Hoxd10* gene in *cis* with the *HoxD*^*Afc*^ allele.

**Results:**

Females carrying this novel *HoxD*^*Del(1*–*9)d10hd*^ allele no longer mutilate males. We conclude that a brain malfunction leading to a severe pathological behavior can be caused by the mere binding to DNA of a transcription factor expressed ectopically. We also show that in *HoxD*^*Afc*^ mice*, Hoxd10* was expressed in cells containing glutamate decarboxylase (*Gad1)* and Cholecystokinin (*Cck*) transcripts, corroborating our proposal that a small fraction of GABAergic neurons in adult hippocampus may participate to some aspects of female courtship.

## Introduction

Although the heterozygous *HoxD*^*Afc*^ genotype proved semi-lethal in both sexes, only sexually mature females displayed an aberrant courtship behavior. When placed with a male for mating, and regardless of the male genotype (i.e. *HoxD*^*Afc*^ heterozygous or wildtype), females repeatedly bit and injured the male’s penises, often up to their complete ablation. In such adult *HoxD*^*Afc*^ heterozygous mice, ectopic *Hoxd10* transcript accumulation was found in numerous scattered cells in the hippocampus [1], while *Hox* genes are never expressed rostral to the hindbrain and its derivatives [2].

## Main text

### Methods

The detailed protocol for the derivation of the CRISPR/Cas9 induced *HoxD*^*Del(1*–*9)d10hd*^ allele was described in [3]. Briefly, the *Hoxd10* CRISPR/Cas9 allele was produced by pronuclear injection of the pX330:hSpCas9 (Addgene ID 42230) vector with the AGAGCGTTAACCTCACCGAC guide sequence oligo cloned as recommended. A small deletion occurred initiating in the guide sequence that removed 10 nucleotides (CCGACAGGCA) corresponding to *mm10* positions chr2: 74695262–74695271. The deletion predicted a *HOXD10* protein product with a truncated homeodomain after position 40. Breeding over more than three filial generations indicated that the *indel* segregated with the *HoxD*^*Del(1*–*9)*^ deficiency. Both *HoxD*^*Del(1*–*9)d10hd*^, *HoxD*^*Del(1*–*9)*^ and *HoxD*^*Del(4*–*9)*^ stocks were maintained by serial backcrosses to (B6CBA)F1 females. Experimental adult females were 12–24 weeks of age.

For in situ hybridization analyzes, freshly dissected brains were mounted in the Optimal cutting temperature (OCT) compound and stored at − 80 °C. In most experiments, pairs of hemi-brains of *HoxD*^*Afc*^ and *HoxD*^(*Del4*–*9)*^ heterozygous or wild type control adult females were mounted in the same block, cut, collected on the same slides and processed together to allow for direct comparison of the *Hoxd10* signals under identical conditions. Usually four parallel sub-series of 14 µm thick coronal cryo-sections were collected, air-dried and stored at − 80 °C. One of the sub-series was stained with Cresyl violet and the position of the sections along the Coronal Allen Brain Atlas was determined. On the day of hybridization, slides were thawed, air-dried and fixed in 4% paraformaldehyde in PBS. In situ hybridizations were carried out at 63.5 °C overnight, followed by stringency washes at 61 °C. The binding of the antisense probe was revealed either by the NBT/BCIP alkaline phosphatase substrate (e.g. Allen Brain Institute http://mouse.brain-map.org/gene), or with the FASTRED alkaline phosphatase substrate (Sigma, SIG-31072) to detect DIG labeled probes, and the Tyramide amplification procedure (PerkinElmer SAT700001EA), followed by 1:100 dilution of Streptavidin Alexa Fluor 488 conjugate (Invitrogene S32354 to detect Fluorescein labeled probes.

*Gad1* and *Cck* antisense riboprobes were synthesized using cDNA plasmid clones as templates (http://www.imagenes-bio.de). Briefly, mouse *Gad1* cDNA clone IRAKp961I2154Q was linearized with Kpn1 and transcribed by T7 polymerase (Promega, #P2075). Mouse *Cck* cDNA clone IRAVp968E034D was linearized with EcoRI and transcribed with T3 polymerase (Promega, #P2083). Mouse *Hoxd10* cDNA clone [1] was digested with *Eco*RI and transcribed by T7 polymerase. Labeled nucleotides were incorporated using digoxigenin (DIG) RNA Labeling Mix (Roche 1122707390), or Fluorescein RNA Labeling Mix (Roche 11685619910). We successfully detected *Hoxd10* with DIG, yet not when a fluorescein labeled antisense cRNA probe was used. This may reflect a higher sensitivity of the alkaline phosphatase enzymatic reaction, which was also supported by the easier detection of the *Gad1* and *Cck* signals with DIG/FAST RED, as compared to the fluorescein/Tyramide enhancement. In double fluorescent in situ hybridization (FISH) experiments *Hoxd10* specific red signal was scored at probe concentrations, when red stained cellular profiles were detected only in the *HoxD*^*Afc*^, and not in either control samples, indicating that conditions were appropriate for specific detection of *Hoxd10* transcripts.

**Fig. 1:**
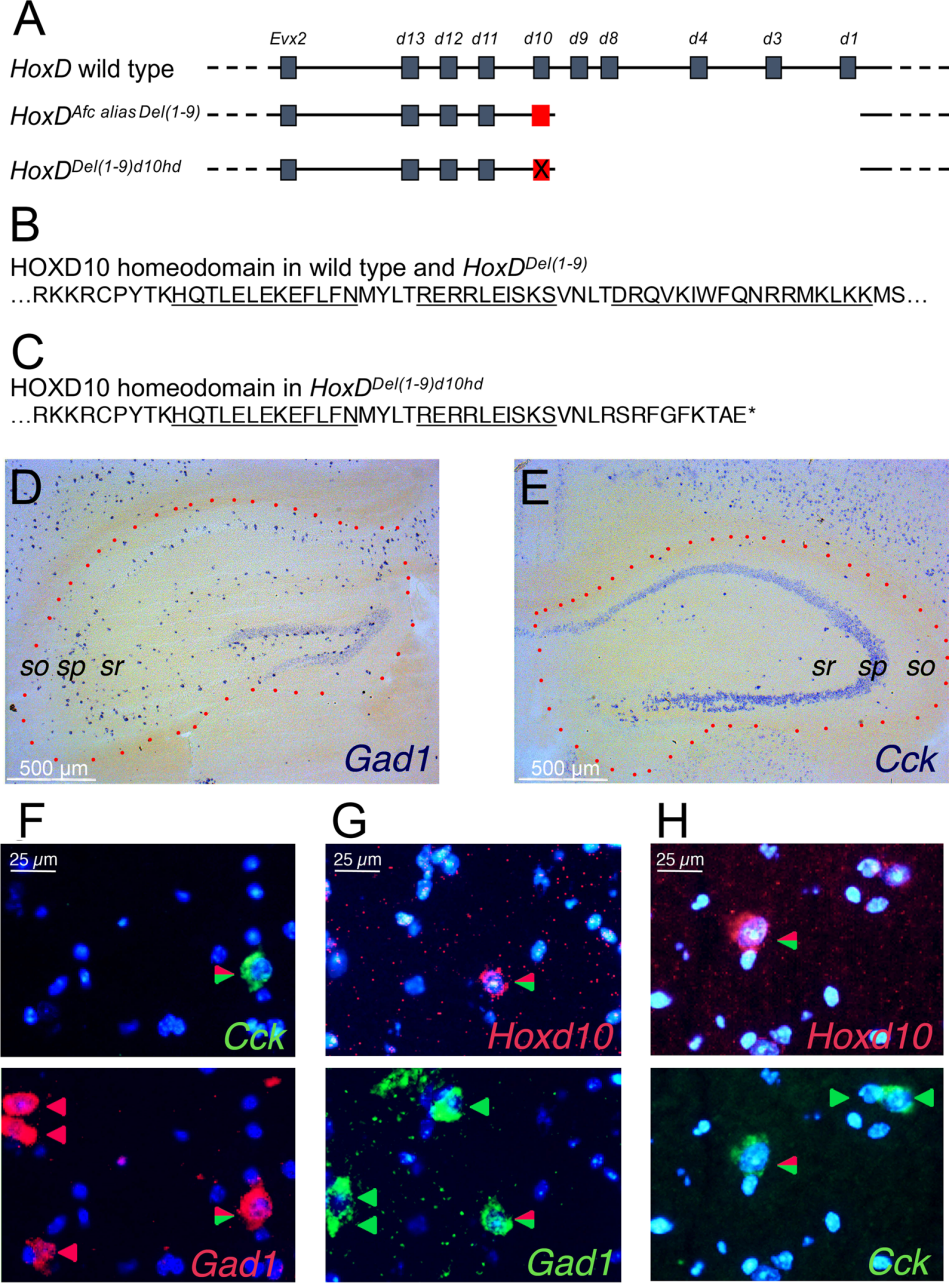
Inactivating mutation in HOXD10 ectopically expressed in a minor GABAergic subpopulation in adult *HoxD*^*Afc*^ brain. **a** Comparison of wild type *HoxD* and the *HoxD*^*Afc*^ *alias Del(1*–*9)* and *HoxD*^*Del(1*–*9)d10hd*^ mutant alleles. Discontinuity of the horizontal line indicates the absence of the genomic segment and the red X indicates the position of the CRISPR/Cas9 hit in the *Hoxd10* homeobox, leading to the generation of the *HoxD*^*Del(1*–*9)d10hd*^ allele. **b** Amino-acid sequence of the HOXD10 homeodomain in both the wild type and the *HoxD*^*Afc*^ alleles. The three alpha helical subdomains are underlined. **c** Amino-acid sequence of the homeodomain in the truncated HOXD10hd protein product. The sequence of the remaining two alpha helical subdomains are underlined and an asterisk indicates an out of frame stop codon. **d**, **e** Details of representative coronal sections of heterozygous *HoxD*^*Afc*^ female brains. The contours of the hippocampal formation are indicated by red dots and three landmark cytoarchirectonic layers are annotated (sr, sp, so, for *strata radiatum*, *pyramidale* and *oriens*, respectively). **d** *Gad1* specific antisense probe reveals positive cells distributed in all layers of Cornu Ammonis (CA). **e** A *Cck* specific antisense probe shows few strongly stained cells in all layers of CA, and a relatively weaker signal in the rest of the cells located in *sp*. **f**, **g**, **h** Simultaneous fluorescent in situ hybridization. Nuclei are shown in blue. **f** Expression of *Cck* in green (top) is detected in one of four *Gad1* positive cells shown below in red (bottom) in CA3 *sr*. **g** Expression of *Hoxd10* (red, top) in one of four *Gad1* positive cells (green, bottom) in CA3 *so*. **h** Expression of *Hoxd10* in (red, top) in one of the three *Cck* positive cells (green, bottom) in CA1 *so*

The double FISH procedure was carried out as in [4]. Pictures were taken with HBO 100 illumination using the appropriate filter sets to visualize red, green and blue fluorescence signals (set 43, 10 and 49 respectively), on a Zeiss Axioplan 2 microscope (Fig. 1f–h). *Hoxd10* red hybridization signals were accepted as positive if the signal could be seen with a 5×/0.25 n.a. 0.17 Zeiss FLUAR objective using filter set 43. Upon higher magnification, a clear cytoplasm signal zone included a negative zone corresponding to the position of a cell nucleus (perikaryon). Images were taken with a Leica DFC300 FX digital color camera. Brightness and contrast were adjusted in Photoshop CS3. Red and blue or green and blue double color images were generated using the HDR2 plug-in.

*Gad1/Cck* colocalizations were obtained from 3 pairs of brains, 14 sections from 3 independent hybridization reactions: 431 cells were *Gad1* positive in the Cornu Ammonis (CA), of which 89 were Gad1/Cck double positive. *Hoxd10/Gad1* colocalizations were obtained from one pair of brains, seven sections from five independent hybridization reactions: 131 cells were *Gad1* positive in CA of which 5 were *Gad1/Hoxd10* double positive. The difference in incidences of *Hoxd10* over total *Gad1* (prop 1 = 0.03816794) and *Cck* over total *Gad1* (prop 2 = 0.20649652) were statistically significant (p = 1.149e−05, 2-sample test for equality of proportions with continuity correction). *Cck/Hoxd10* colocalizations were obtained from one pair of brains, five sections from two independent hybridization reactions: 39 cells were *Cck* positive in CA, of which 9 were *Cck/Hoxd10* double positive. The difference in incidences of *Hoxd10* over total *Gad1* (prop 1 = 0.03816794) and *Hoxd10* over total *Cck* (prop 3 = 0.23076923) were statistically significant (p = 0.0004497, 2-sample test for equality of proportions with continuity correction). In all cases, any *Hoxd10* positive cells proved positive for either *Gad1* or *Cck* depending on the probe mix under investigation. The product of prop2 and prop3 was in good agreement with prop1 (0.046 vs 0.038), therefore we concluded that further technical repetitions with these same techniques were unlikely to bring additional information.

### Results

To confirm the causal role of *Hoxd10* ectopic expression in this unusual behavior, we induced a deletion in the homeobox of the *Hoxd10* gene *in cis* with the *HoxD*^*Afc*^ allele as a secondary mutation (Fig. 1a). Non-homologous end joining of genomic DNA after exposure to a single guide RNA and the Cas9 endonuclease in fertilized eggs resulted in a 10 base-pair long deficiency in the *Hoxd10* homeobox, giving rise to the *HoxD*^*Del(1*–*9)d10hd*^ allele. This mutant allele had lost the third alpha-helix of the *HOXD10* homeodomain necessary for the binding of this transcription factor to its DNA target sites (Fig. 1b), due to a protein truncation from the 40th position of the homeodomain onwards, replacing 34 residues by a 10 residues frameshifted sequence (Fig. 1c).

We crossed this allele out through three consecutive generations and observed twelve adult females caged with males. Studs were followed for the appearance of injuries at their external genitals. Heterozygous *HoxD*^*Del(1*–*9)d10hd*^ females bred successfully, without any indications of atypical female courtship (0 out of 12). This was in marked contrast with the observation of 12 out of 18 *HoxD*^*Afc*^ females carrying the intact *Hoxd10* homeobox sequence and showing genital biting [1]. This difference was statistically significant (p = 0.001071) by the 2-sample test for equality of proportions with continuity correction Other abnormal phenotypic traits associated with the *HoxD*^*Afc*^ allele, like malocclusion and slow postnatal weight gain were also rescued [3]. These results provide strong genetic evidence of the direct role of the *HOXD10* transcription factor in bringing about the courtship aberration observed in *HoxD*^*Afc*^ mice.

This atypical female courtship anomaly occurred in animals with a low abundance of *Hoxd10* positive cells in adult forebrain, in both sides and at any observed rostra-caudal sections of the hippocampal formation, which display molecular and neuroanatomical characteristics reminiscent of a small subpopulation of GABAergic interneurons [1, 5], as characterized by the detection of both the *Gad1* and *Cck* markers (Fig. 1d, e). Double labeling simultaneous FISH analyses with *Hoxd10*-*dig* and *Gad1*-*fluo* pair of probes indeed showed *Hoxd10* positive cells localized selectively in the hippocampus, distributed in any of the layers of the Cornu Ammonis (CA) fields where it co-localized with *Gad1* (Fig. 1f–h). Furthermore, by using *Cck*-*flou* and *Hoxd10*-*dig* probes simultaneously, we scored the *Hoxd10* specific red signal in cells accumulating *Cck* transcripts (Fig. 1h). As all *Cck* positive non-principal cells seemed included in the *Gad1* labeled pool, and since all *Hoxd10* positive cells were part of the *Cck* positive non-principal pool, we concluded that ectopic *Hoxd10* transcripts accumulated in a very sparse subpopulation of *Cck* positive GABAergic cells. Of note, *Hoxd10* like other *Hox* genes is not expressed in any cells of a normal adult forebrain [6].

### Discussion

The *HoxD*^*Afc*^ phenotype followed a gender-specific pattern of expressivity, limited to sexually receptive females, despite the fact that ectopic expression of *Hoxd10* was similar in both sexes. The ectopic presence of this HOX product in CCK positive GABAergic neurons in adult hippocampus may thus interfere with the implementation of a particular genetic program in a sexually dimorphic manner, perhaps through the property of such proteins to exert a dominant negative effect in various contexts [7]. CCK signaling was previously associated with a sex-dependent control of behavior and its level seems to be modulated during the estrus cycle [8]. Also, the inactivation of the Cck2 receptor, which presumably mediates some effects of CCK neuropeptides in postsynaptic neurons, elicits behavioral alterations distinct in females as compared to males [9]. Altogether, this is consistent with a gender-specific role of CCK positive GABAergic cells in the modulation of behavior [10]. A persistent ectopic expression of *HOXD10* in CCK positive hippocampal GABAergic cells may thus interfere with the function of these cells in controlling the dynamic physiological status of females during the estrous cycle [11].

### Limitations

The Identification of GABAergic cells and the co-localization of ectopic *Hoxd10* gene product accumulation was carried out relying on in situ hybridization detection of mRNA. This methodology provided a way to circumvent protein-based localization assays due to the absence of the required high-quality antibody. However, this approach does not allow for a rigorous evaluation of the *HOXD10* protein distribution.

## Abbreviations

*HoxD*^*Afc*^: *Atypical female courtship* allele of the *HoxD* locus
*Gad1*: glutamate decarboxylase
*Cck*: cholecystokinin
CA: Cornu Ammonis
FISH: fluorescent in situ hybridization
OCT: optimal cutting temperature
DIG: digoxigenin
